# Development of Environment-Friendly Membrane for Oily Industrial Wastewater Filtration

**DOI:** 10.3390/membranes11080614

**Published:** 2021-08-11

**Authors:** Mohammed Alquraish, Yong Tzyy Jeng, Mohamed Kchaou, Yamuna Munusamy, Khaled Abuhasel

**Affiliations:** 1 Department of Mechanical Engineering, College of Engineering, University of Bisha, Bisha 67714, Saudi Arabia; malqraish@ub.edu.sa (M.A.); kabuhasel@ub.edu.sa (K.A.); 2 Department of Petrochemical Eng, Faculty of Engineering and Green Technology, Universiti Tunku Abdul Rahman, Kampar 31900, Malaysia; yongtj@utar.edu.my (Y.T.J.); yamunam@utar.edu.my (Y.M.)

**Keywords:** membrane filtration, oil-water separation, oil rejection rate, performance

## Abstract

Latex phase blending and crosslinking method was used in this research work to produce nitrile butadiene rubber-graphene oxide (NBR-GO) membranes. This fabrication technique is new and yields environmentally friendly membranes for oil-water separation. GO loading was varied from 0.5 to 2.0 part per hundred-part rubber (pphr) to study its effect on the performance of NBR-GO membrane. GO was found to alter the surface morphology of the NBR matrix by introducing creases and fold on its surface, which then increases the permeation flux and rejection rate efficiency of the membrane. X-Ray diffraction analysis proves that GO was well dispersed in the membrane due to the non-existence of GO fingerprint diffraction peak at 2θ value of 10–12° in the membrane samples. The membrane filled with 2.0 pphr GO has the capability to permeate 7688.54 Lm^−2^ h^−1^ water at operating pressure of 0.3 bar with the corresponding rejection rate of oil recorded at 94.89%. As the GO loading increases from 0.5 to 2.0 pphr, fouling on the membrane surface also increases from Rt value of 45.03% to 87.96%. However, 100% recovery on membrane performance could be achieved by chemical backwashing.

## 1. Introduction

In 2015, the United Nations (UN) General Assembly recognized clean water—a critical element for all living things on this planet—as one of the sustainable development goals (SDG). However, more than eight hundred children under the age of five die every single day from water contamination, pollution, and poor sanitation. According to the World Water Council, 700 million humans will still live in water scarcity by the year 2030 [1]. This ecological crisis is the consequence of the rapid urbanization and industrialization that directly causes water pollution through the discharge of large volumes of toxic pollutants such as oils, organic solvents, dyes, toxic chemicals, microplastics, biological substances, and heavy metal ions into water body [2,3]. Thence, the development of effective technologies to protect our ecosystem is greatly needed at this point of time. Traditional oil from water separation methods, such as coagulation, dissolved air floatation, gravity separation, flocculation, and de-emulsification have some drawbacks, for example high operation cost, low filtration efficiency, energy consuming, complex processes, corrosion of the equipment, and recontamination. Moreover, these techniques are ineffective in separation of micron size oil in emulsion form [4,5]. Consequently, the membrane separation method has been widely recognized as an alternate approach because of its excellent flexibility, cost effectiveness, operational easiness, fairly high efficiency, and environmental benevolence [6,7]. Regrettably, the major barrier for concrete application of membrane technology in oily wastewater filtration is the fouling tendency of membrane, the trade-off effect between permeation flux, and the rejection efficiency and poor chemical resistance [8].

In recent times, many scholars worked expansively on ultrafiltration (UF) membranes to find a solution for the trade-off problem during filtration. For instance, Ahmad et al. [9] fabricated UF membrane using polyvinyl chloride mixed with salt-induced pluronic F127 and bentonite to solve the trade-off issue. In this work, water flux of 607.8 Lm^−2^ h^−1^ and oil rejection rate of more than 92.8% were achieved. Ma et al. [10] employed a polyimide membrane using zeolitic imidazolate framework-8@thiolated graphene (ZIF-8GSH) to overcome this problem. This membrane could self-clean to improve permeation flux and exhibit an oil rejection rate of 99%. Thus, NBR-GO membrane was also produced to overcome the trade-off problem. NBR latex was selected for membrane application because of its remarkable structural reliability and chemical resistance [11]. The mechanical properties and chemical resistance of NBR could be altered using the three-dimensional sulphide linkages in the crosslink network. However, the hydrophobic profile of NBR latex that can compromise the permeation flux is still the foremost concern in membrane filtration technology. Therefore, our aim is to introduce water affinity into the NBR membrane by incorporating GO with oxygen functional groups, such as epoxy, carboxyl, carbonyl, and hydroxyl, on its basal planes and sheet edges [12,13,14]. Apart from that, GO was proposed for membrane fabrication due to its distinctive properties, such as a high surface area, hydrophilic nature, good compatibility with polymers [15,16], increased resistance towards fouling, improved separation efficiency, and ability to impart excellent tensile strength to the membrane [17,18]. In the work done by Alammar et al., incorporation of 1 wt% GO into polybenzimidazole membrane had caused oil rejection up to 99.9% with permeation flux of 91 L m^−2^ h^−1^ bar^−1^.The membrane was prepared using a phase inversion method with dimethylacetamide as solvent [19]. The addition of GO from 0.05 to 0.2 wt% in the polysulfone membrane improved water permeability by 97% with oil rejection up to 97.9%. The improvement in water permeability is achieved by reduction of water contact angle from 77° due to the hydrophobic nature of polysulfone to around 65° [20]. In another work using polyvinyl chloride as membrane material, the addition of GO imparted hydrophilicity to the membrane by reducing the contact angle of the membrane with water. The reduction in contact angle with incorporation of GO is due to the large amount of hydrophilic functional groups in GO, which enhances the affinity of PVC membranes towards water molecules [21].

There are plenty of reported methods in the literature to produce polymer-based membranes. For instance, stretching [22], sintering [23,24], phase inversion [25,26], and tracking etching [27,28]. However, all these techniques are time consuming, involve many steps, yield poor dispersion of GO, and might include large usage of non-environmentally friendly solvents, such as methyl ether ketone (MEK), tetrahydrofuran (THF), and chlorobenzene [29,30,31]. Gao et al. had produced cellulose modified polyvinylidene fluoride membrane using a simple, facile, and environmentally friendly method but nearly 10 h of laboratory preparation time is required for the membrane, which includes chemical preparations, preparation of dope solutions, coatings, and drying [32]. Therefore, a cost effective, sustainable, and environmentally friendly method is required for commercial production of polymer membrane [33].

In this research, NBR-GO membranes were produced through latex phase blending and crosslinking method, whereby the membrane could be produced in less than three hours in the laboratory through a water-based process. GO is anticipated to disperse well because the blending is carried out in the aqueous phase of the emulsion of latex. It could further promote the dispersion of hydrophilic functional groups containing GO.

Fabrication of membrane using latex phase blending and crosslinking is a new method and thus each parameter involved needs to be studied to optimize membrane performance. Therefore, the effect of GO loading on tensile properties, morphology, and performance of the membrane is evaluated and reported in this paper. These findings are crucial for optimized practical application of NBR-GO membrane in oily wastewater separation.

## 2. Materials and Methods

### 2.1. Materials

NBR latex was supplied by Synthomer, Kulang, Johor, Malaysia. The total solid content (TSC) was calculated to be 65.70% and the molecular weight is 17.20 × 10^4^ g/mol. Crosslinking chemicals such as sulphur (S), zinc oxide (ZnO), zinc mercaptobenzothiazole (ZMBT), and zinc diethyldithiocarbamate (ZDEC) were in the slurry form with TSC values of 49.41%, 56.02%, 54.86%, and 54.42% respectively. All these crosslink chemicals were supplied by Zarm Scientific & Supplies, Bukit Mertajam, Penang Malaysia. Graphene oxide (GO) was purchased from GO Advanced Solutions, Bangi, Selangor, Malaysia. A single layer of GO has an average length of 873.5 ± 141.1 nm and thickness of 1–2 nm.

### 2.2. Production of NBR-GO Membranes

Firstly, GO suspension with TSC value of 2% was prepared by mixing 0.2 g of GO with 10 g of deionized water. The suspension was ultra-sonicated for one hour with a probe-type QSonica ultrasonic homogenizer model Q500 supplied by Gaia Scientific, Puchong, Selangor, Malaysia. This step is carried out to separate the GO sheets that tend to adhere to each other and form aggregates. Weak Van der Waals forces hold the GO sheets in an ordered, stacked structure. A homogenous slurry was obtained and used in latex compounding for membrane production. Selected quantities of blending recipes in parts per hundred-part of rubber (pphr) consisting of 100 pphr NBR latex, 1.0 pphr of ZnO and ZDEC each, 0.5 pphr ZMBT, GO loading varied as 0.5, 1.0, 1.5, and 2.0 pphr, and lastly, 1.0 pphr sulphur was added into a beaker. The amount of compounding chemicals was chosen based on previous reported works on NBR latex film productions [34,35]. The compound was then stirred at a speed of 350 rpm for 30 min with IKA overhead stirrer model EUROSTAR Digital 20 supplied by IKA Works Asia, Rawang, Selangor, Malaysia. The compounds were casted into NBR-GO membranes with the thickness of 0.1 mm on glass plates with a laboratory size membrane auto casting machine, model A4K-S564 from Autonics, Malaysia. A distance of 220 mm and a forward speed of 150 rpm was used to cast the membranes. The casted membranes on the glass plate were crosslinked in a conventional oven model Memmert supplied by Interscience, Shah Alam, Selangor, Malaysia at temperature 100 °C for 2 h. The formulation used for membrane production and its designations are summarized in Table 1.

### 2.3. Characterization of the Membranes

The membrane’s surface morphology was observed under a field emission scanning electron microscope (FESEM), model JEOL JSM 6710F, supplied by JEOL USA Inc., Peabody, MA, USA. In order to enhance the electron charging during image processing, all the membranes were sputter-coated with platinum particles prior to scanning. Functional groups on the membrane surface were determined using Fourier-transform infrared (FTIR) spectroscopy. The analysis was carried out using attenuated total reflectance (ATR) technique at room temperature between the wavelength of 400 and 4000 cm^−1^ using Spectrum RX1 Perkin Elmer Analyzer supplied by Perkin Elmer Sdn. Bhd Petaling Jaya, Selangor, Malaysia. Dispersion of GO in the membranes was characterized using X-ray diffraction (XRD) analysis using Siemens XRD Diffractometer Model 5000 supplied by DKSH Technology Sdn. Bhd., Petaling Jaya, Selangor, Malaysia. The diffractometer operates using nickel-filtered copper K_α_ radiation with λ = 0.154 nm. Parameters of the analysis includes step scan with scanning rate of 2°/min between the scanning angles of 10 and 60°. Crosslink density of each membrane were determined using the methodology in our previous publication [36]. Five samples were used for each membrane category and an average crosslink density value was calculated from the samples.

### 2.4. Performance Studies

The ultimate tensile strength (UTS), E-modulus (E), and percentage of elongation at break (%EB) of the membranes were determined using the tensile test. For each membrane composition, seven dumbbell-shaped samples were prepared, and the average value of each test result was calculated. Each sample had a gauge length of 26 mm and a neck width of 3 mm. Tensile test was performed following ASTM D882-10 standard, with a load setting of 450 N and a pulling speed of 100 mm/min using a light-weight tensile tester, model H10KS-0748 from Tinius Olsen Ltd., Redhill, UK, supplied by Leader Technology Scientific (M) Sdn. Bhd., Seri Kembangan, Selangor, Malaysia.

Dead-end membrane test rig supplied by Shxp Trading, Bandar Tasek Selatan, Selangor, Malaysia, was used to carry out the permeation flux test at the operating pressure between 0.1–0.3 bar. Emulsified oily wastewater with an oil content of 1000 ppm was prepared in the laboratory by emulsifying diesel with 0.01 wt% of sodium dodecyl sulphate (SDS) in deionized water for 30 min using a probe-type QSonica ultrasonic homogenizer model Q500. The size of the oil particles was determined using a Lighthouse liquid particle counter, model LS-20 supplied by Lighthouse Worldwide Solution (M) Sdn. Bhd., George Town, Penang, Malaysia. The size fell within a range of 1.0–50 µm with the majority of particle size is at 6 µm. The permeation flux was calculated using Equation (1). Thirty mL permeate was collected for each run and the time taken was recorded. The diameter of the membranes used for filtration cell was fixed to 50 mm to provide effective area for permeation of 1.0179 × 10^−3^ m^2^. Five membrane samples from each category were used for the test and the average permeation flux was calculated.

(1) $$J=\frac{V_{p}}{A\times t}$$

where:

J = Permeation flux, $\frac{L}{m^{2}\cdot hr}$
$V_{p}\;$= Permeate volume collected, L
A = Effective membrane area, $m^{2}$
*t* = Time taken to collect the measured volume of permeate, hr

Oil rejection efficiency of the membranes was assessed by performing chemical oxygen demand (COD) analysis on the filtrate from permeation flux test. Five filtrate samples were used for each membrane category. HACH COD vials and a HACH COD digester were used for digestion of the filtrate and the COD value was recorded using HACH DR6000 UV-VIS spectrophotometer. The oil rejection efficiency was then calculated based on Equation (2).

(2) $$R=\left(1-\frac{COD_{p}}{COD_{f}}\right)\times 100\%$$

where:

R = Oil rejection efficiency, %
$COD_{p}$ = COD level in permeate, mg/L
$COD_{f\;}$= COD level in feed, mg/L

The total fouling ratio (*R_t_*) and flux recovery ratio (FRR) of the membranes were determined at the constant operating pressure of 0.1 bar using Equations (3) and (4), respectively [37]. Firstly, water flux for clean deionized water was measured as *J_w_*_1_, the flux of 1000 ppm synthetic oily wastewater as *J_p_*; after that, the membrane was cleaned by rinsing with clean water at room temperature and the water flux was recorded as *J_w_*_2_. For comparison, the backflushing technique was also applied in the membrane cleaning for 1 min [38]. These values are an indicator for the antifouling performance. Membranes with low *R_t_* and high FRR values would have excellent fouling resistance. Five samples were used for each membrane category and the average *R_t_* and FRR values had been reported.

(3) $$R_{t}=\left(\frac{J_{w1}-J_{p}}{J_{w1}}\right)\times 100\%$$

(4) $$FRR=\left(\frac{\;J_{w2}}{J_{w1}}\;\right)\times 100\%$$

where:

$J_{p}$ = Permeation flux of synthetic oily wastewater
$J_{w1}$ = Permeation flux of deionized water
$J_{w2}$ = Permeation flux of deionized water after membrane cleaning

## 3. Results

### 3.1. Characterization of the Membrane

**Figure 1:**
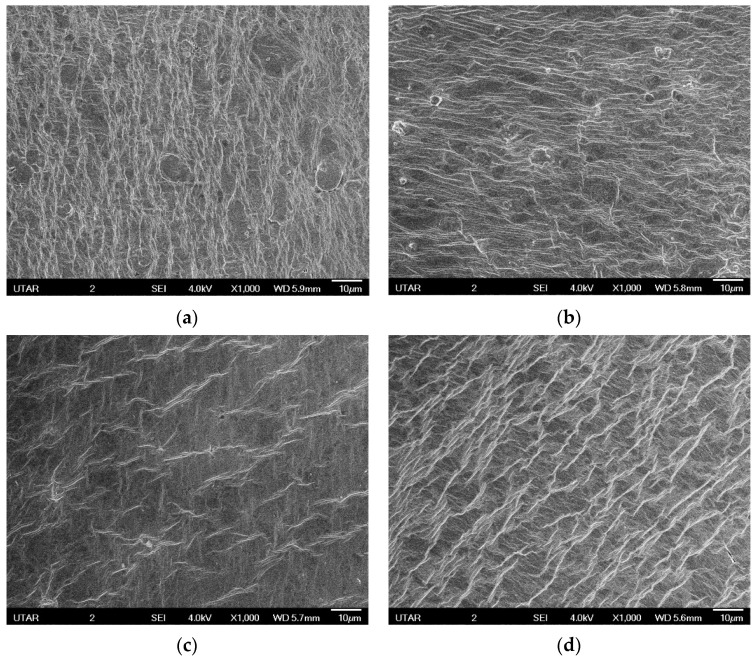
SEM observations of the NBR-GO membrane surfaces. (**a**) 0.5 pphr GO. (**b**) 1.0 pphr GO. (**c**) 1.5 pphr GO. (**d**) 2.0 pphr GO.

NBR-GO membranes demonstrate a clear wrinkled and folded structure as shown in Figure 1. This kind of structure is attributed to the unevenly distributed oxygen functional groups of GO that produce the folding and notching effect of GO sheets edges on the NBR membrane surface during casting. The low-density GO moves up towards the membrane surface during the crosslinking process. Ridged, scrolled, and creased surface on the membranes create folds, edges, and channels which can increase the effective surface area for oily wastewater UF. Consequently, improvement in permeation flux and oil rejection efficiency are expected. Creation of folds on the membrane surfaces filled with GO were also reported in other works [2,39,40,41]. Some macropores could also be observed on the surfaces of membranes with 0.5 and 1.0 pphr GO loadings. These pores could not be observed on membranes with higher GO loadings because the surfaces are covered with GO. The existence of pores also did not increase the water flux compared to membranes with 1.5 and 2.0 pphr GO loading which indicates the dominance of GO nanosheets surface structures and hydrophilicity on water molecule affinity and permeability.

**Figure 2:**
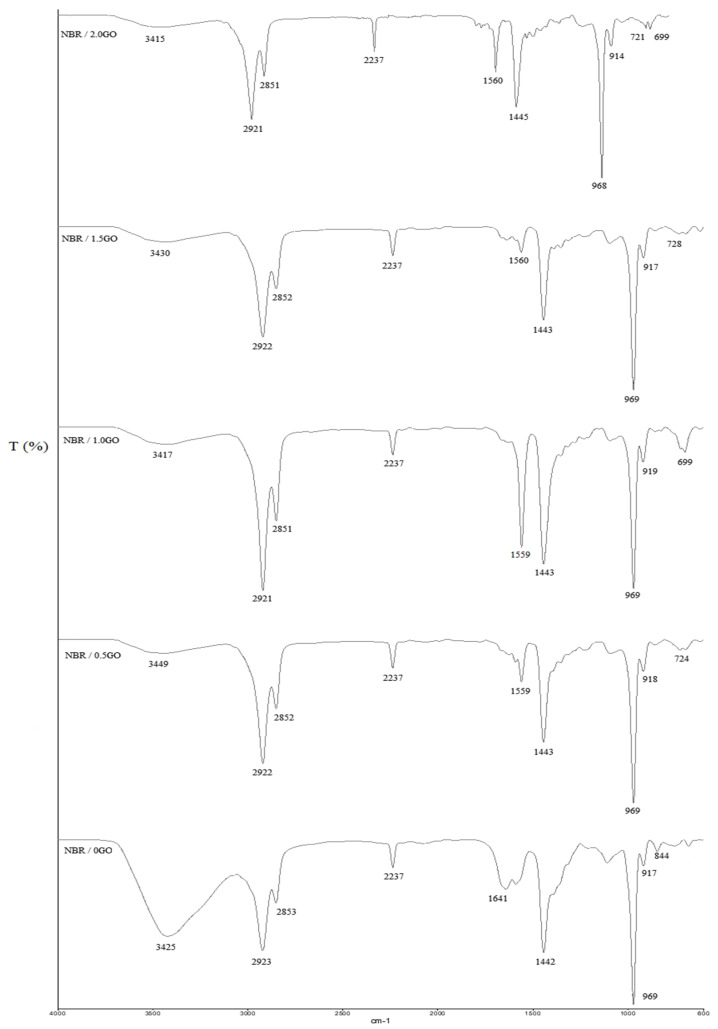
FTIR spectra for pure NBR and NBR-GO membranes at different GO loadings.

The creases and folds are getting more prominent as the GO loading increases and this has proven more movement of GO sheets to the surface of the membrane during casting and crosslinking steps. The channels between the ridges on the membrane can allow the oil particles to be trapped in the grooves of the membrane, and hence, high oil rejection could be achieved. However, as the GO loading increases, more wrinkles and ridges directly cause the groove width to be narrower, consequently, the bigger oil droplets are likely to recess and clog the membrane. As such, the higher possibility for membrane fouling occurs with higher GO loading. On the other hand, GO, which acts as the additives in the UF membrane, is hydrophilic in nature due to its oxygen functional group, and hence, this significantly increases the permeation flux performance as the GO loading increases. Figure 2 presents the FTIR spectrum of NBR and the NBR-GO membranes.

The peak at 3400 cm^−1^ indicates the existence of the O–H functional group from the surface-bounded H_2_O molecules. The peaks at 2237 cm^−1^, 1641 cm^−1^, and 1559 cm^−1^ are apportioned to the C≡N groups, C=C bonds and N–H bending of NBR, respectively [42]. The peak at 1442 cm^−1^ confirms the occurrence of carboxylic acid O-H stretching while C-O bending is denoted by 969 cm^−1^ peak [43]. The bands between 917 cm^−1^ to 699 cm^−1^ specify the occurrence of S–OR and S–S disulphide stretching of sulphur crosslinked system in the NBR polymer matrix. No new peak was observed; thus, it can be deduced that the NBR and GO are interacted physically and not chemically. The NBR polymer chains could be adsorbed or entrapped on the surface of GO which creates a good filler -matrix interphase.

**Figure 3:**
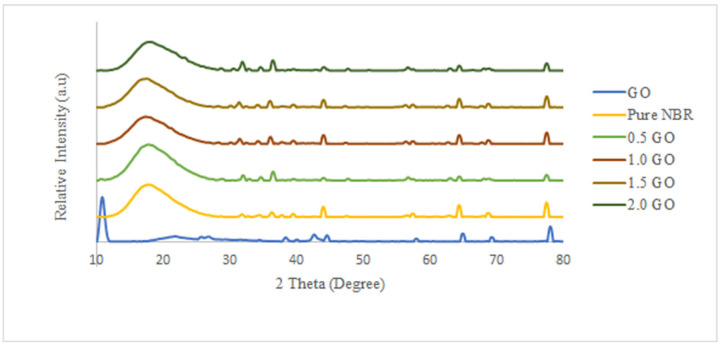
XRD pattern of GO, Pure NBR, and NBR-GO membranes at different GO loadings.

XRD analysis was performed to evaluate the dispersions of GO nanosheets in the NBR membrane and presented in Figure 3. The ordered intercalated structure of GO nanosheets is represented by the sharp and strong peak found at 2θ value of 10–12° which is the characteristic diffraction peak for GO [44]. The GO nanosheets are intercalated due to the presence of oxygen functional groups such as carboxyl, carbonyl, epoxide, and hydroxyl which adhere to the edges and basal planes of GO sheets. Typically, 6–7 graphene layers will heap together to form the GO nanostructure. A broad diffraction peak is shown in the pure NBR membrane, representing that the pure NBR is a non-crystalline structure. For the XRD diffraction of all NBR/GO membranes, no GO characteristic peak could be distinguished, and it can be concluded that there is no layer-by-layer restack of GO in the NBR matrix, thus the homogenous dispersion state of GO in NBR matrix is proposed [45]. NBR polymer chains could penetrate between the GO layers and exfoliate the layers, thus destroying the ordered intercalated structure of GO.

All the membranes contain 1.0 pphr sulphur and are being crosslinked at the same temperature and time duration. Crosslinks form a stable three-dimensional sulphide network between the NBR polymeric chains. These linkages impart high mechanical strength and chemical resistance to the membrane. The crosslink density in each membrane is shown in Table 2. GO loading was found not to influence the crosslink density as the crosslink density between all the samples was almost similar to one another.

### 3.2. Performance Study

#### 3.2.1. Tensile Properties

**Figure 4:**
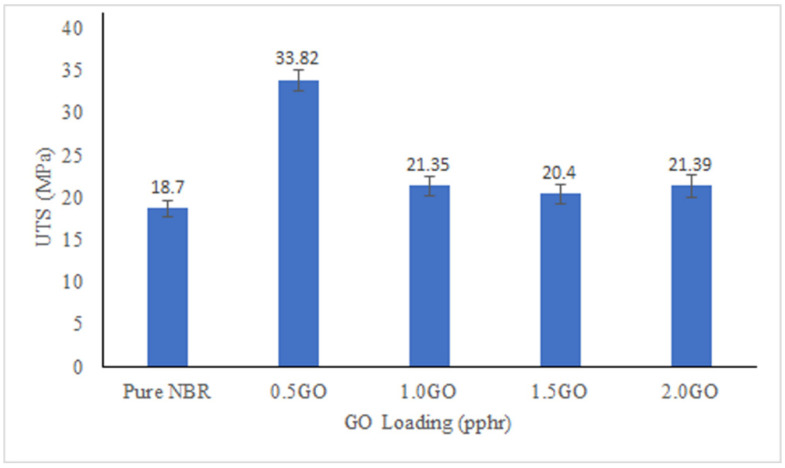
Ultimate tensile strength (UTS) of the membranes with different GO loading.

**Figure 5:**
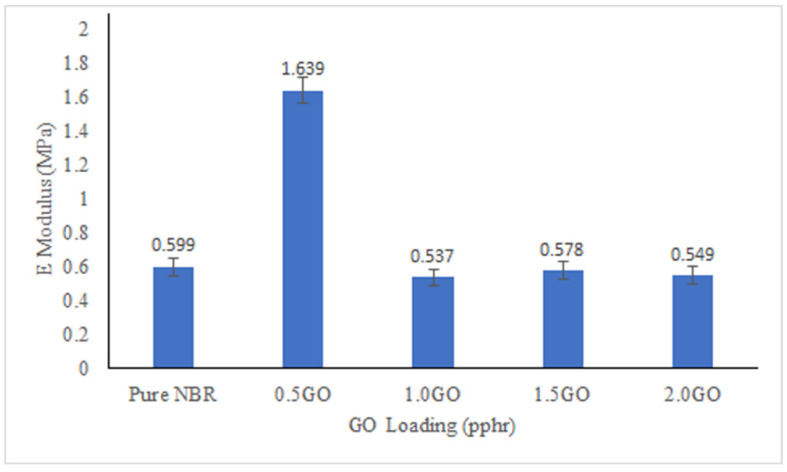
E-modulus of the membranes at different GO loading.

**Figure 6:**
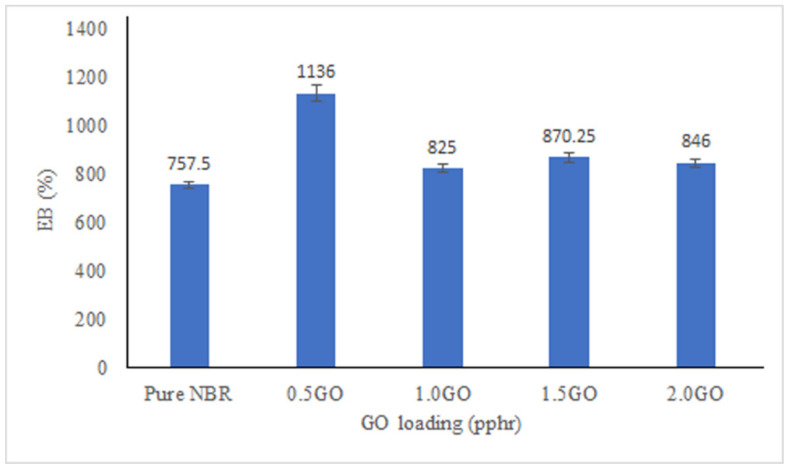
Elongation at break of the membranes at different GO loading.

The UTS, E-modulus and EB% are shown in Figure 4, Figure 5 and Figure 6. All the membranes filled with GO showed higher UTS compared to pure NBR membrane with optimum increment of UTS achieved at 0.5 pphr GO loading. GO has strong mechanical reinforcing effect to the NBR polymer matrix due to excellent interfacial physical interaction between the GO and NBR matrix [46]. This interaction is promoted by the high surface area of dispersed GO sheets. As the GO loading increases, there will more concentration of GO on the surface of the membranes as suggested by the creases and folds in SEM images that caused the demobilization effect of polymeric chain, causing a decrease in UTS of the membranes. The EB% of the membranes show similar trend with UTS. Improvement of EB with addition of fillers confirms strong interfacial interaction between the filler and matrix, thus when subjected to tension the filler and matrix acts as a synergistic system and elongate in the direction of load without detachment of the fillers from the matrix [47]. The modulus of the membrane increases more than 200% at 0.5 pphr GO loading compared to pure NBR but then does not show any significant improvement compared to modulus of pure NBR at GO loading between 1 to 2.0 pphr.

#### 3.2.2. Permeation Flux

**Figure 7:**
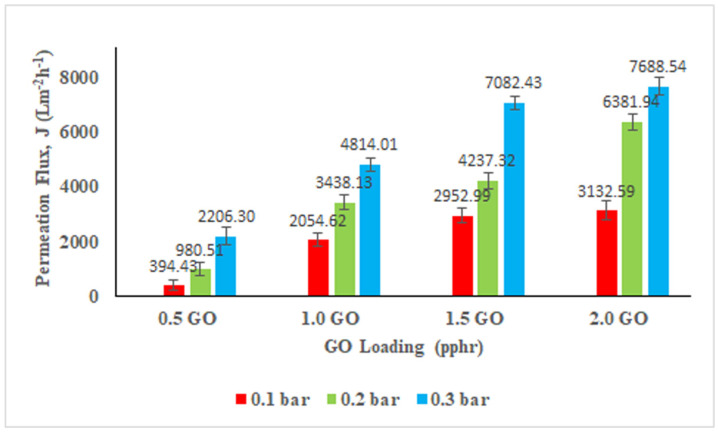
Permeation flux of the membranes tested at different operating pressure.

The permeation flux of NBR-GO membranes strongly relies on its microstructure and morphology. Influence of different GO loading on the permeation flux performance of the membranes is shown in Figure 7. It is understood that the pure NBR membrane did not show any permeation flux because of the hydrophobic characteristic of NBR polymer matrix. Therefore, the inclusion of GO into the NBR matrix results in the permeability of the membranes [48]. As evidenced from the Figure 7, the membranes permeability increases from 394.4.3 to 3132.59 L^2^ m^−2^ h^−1^, 980.51 to 6381.94 L^2^ m^−2^ h^−1^, and 2206.30 to 7688.54 L^2^ m^−2^ h^−1^ at 0.1, 0.2, and 0.3 bar, respectively. This is because the higher the GO loading, the more wrinkles have formed on the membranes as shown by SEM images. This directly generates more effective areas for the UF to occur.

The permeation flux in the system escalates proportionally with increment in operating pressure. At higher value of operating pressure, the water droplets can readily permeate through the membrane because of the higher driving force which pushes the droplets across the membrane [49]. As compared to the other literature, NBR-GO membranes can have a high permeation flux performance at a low operating pressure of 0.1–0.3 bar and this has proven that the membrane is considerably cheaper in terms of energy and cost. Even though the permeation flux of NBR-GO membranes is exceptional, selection of the right composition of the membrane is ultimately depended on the oil rejection efficiency. This performance is critical since the governing body requisite on allowable oil content in treated wastewater to be discharged in water body must be fulfilled.

#### 3.2.3. Oily Rejection Efficiency of NBR-GO Membranes

**Figure 8:**
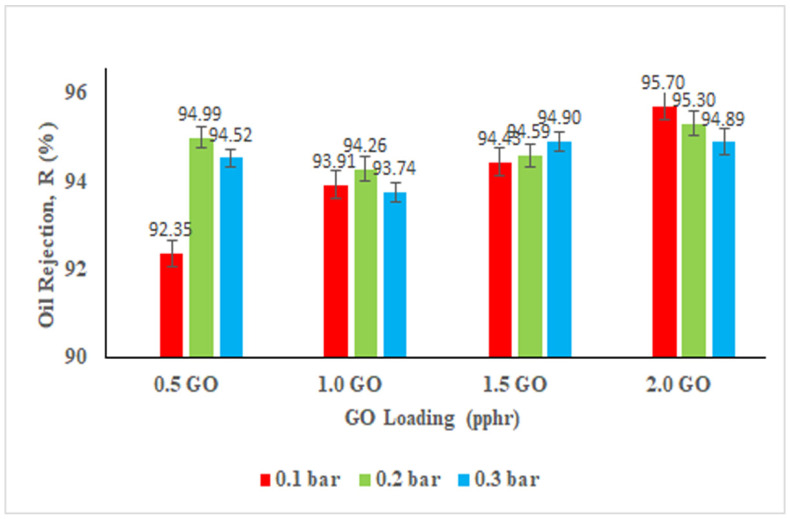
Oil rejection (%) of the membranes at different operating pressures.

Figure 8 shows that the least oily wastewater rejection efficiency for the membranes with different GO was recorded at 92.35%. This high rejection efficiency is mainly attributed to the wrinkles and the grooves between the ridges on the membrane that can trap the oil particles in the grooves of the membrane. As higher operating pressure is being applied, accumulation of oil droplets on the membrane surface starts to happen and eventually a thin layer of oil films will be formed. These will turn to be a blockade for the passage of other oil droplets in the water during filtration. Nevertheless, the relationship between operating pressure and oil rejection efficiency is not proportional [50]. Consequently, 2.0 pphr GO filled NBR membrane is more desirable because it has the highest permeation flux at any operating pressure and high oil rejection efficiency of up to 95.7%. Furthermore, it has successfully fulfilled the global oil discharge limit with the maximum allowable oil concentration of 72 mg/L for any 24-h period set by the Environmental Protection Agency (EPA), the United States [51]. However, in future work the oil rejection could be further improved by modification of GO with more hydrophilic functional groups and formation of coating layer on the surface of the membrane.

The results obtained from this study using the NBR-GO flat sheet membranes are comparable or even better than previous reported research works. One wt% GO filled polyether sulfone flat sheet membrane produced by Junaidi et al., shows rejection rate of oil around 50% and water flux of 95.12 ± 8.51 Lm^−2^ h^−1^ at pressure 1 bar [40]. Meanwhile in another study by Amid et al., 0.25 wt% GO filled polycarbonate flat sheet membrane shows 99% rejection rate of oil with water flux of 85.85 Lm^−2^ h^−1^ at operating pressure of 2 bar [52]. Isotactic propylene-polydopamine-GO membrane shows an oil rejection rate of more than 99% but the corresponding water flux is only recorded at 188 Lm^−2^ h^−1^ at an operating pressure of 0.1 MPa [53].

#### 3.2.4. Antifouling Properties

**Figure 9:**
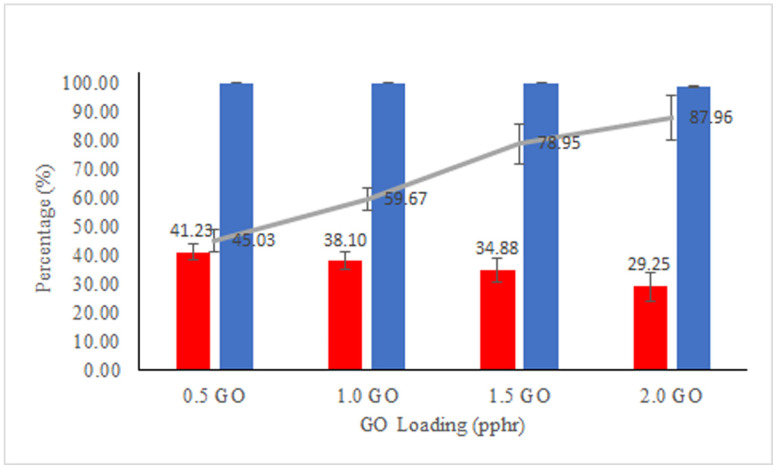
FRR (normal rinsing) 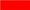, FRR (back flushing) 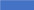, and Rt 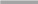 values of the membranes.

The fouling and antifouling performance of the membranes were evaluated by R_t_ and FRR, respectively, after the filtration of the oily wastewater, and the results are shown in Figure 9. It can be found that the 2.0 pphr GO filled membrane has the highest R_t_ of 87.96% and the 0.5 pphr GO filled membrane has the lowest R_t_ of 45.03%. This is due to the foulants in terms of bigger oil particle size and the SDS that can form filter-cake-layers on the membrane, fasten the fouling rate and result in a very serious R_t_. Nevertheless, membrane fouling is very common to all membranes regardless of commercial line or research line.

To confirm that the membranes can be recycled, the antifouling performance through FRR is carried out. As shown in Figure 9, normal rinsing with water at room temperature can only recover 41.23%, 38.10%, 34.88%, and 29.25% for 0.5 GO, 1.0 GO, 1.5 GO, and 2.0 GO filled NBR membrane, respectively. This may be due to the normal washing method that could not wash off the oil particle and the SDS on the membrane fully. On the other hand, the membranes can be fully recovered using the back-flushing method. This method applies higher pressure on the permeate side of the membrane, in so doing causing backward movement of the permeate through the membrane [54]. Backwashing is the furthermost broadly employed fouling reversal technique used in industry [2].

There are so many membrane cleaning methods reported in the literature: pneumatic cleaning [55,56], ultrasonic cleaning [57,58], sponge ball cleaning [59,60], and chemical cleaning [61,62] However, backflushing is the most accessible and cheaper technique to obtain maximum recovery among all. Anyhow, the effect of membrane cleaning methods on the NBR-GO membrane will be evaluated in the next study.

## 4. Conclusions

NBR-GO membranes were produced successfully using the latex compounding and crosslinking method. This production method is water based and requires approximately 2 h laboratory preparation time. NBR membrane with 2.0 pphr GO loading has shown the highest oil rejection efficiency of 95.70% with corresponding permeation flux of 3132.59 Lm^−2^ h^−1^ at pressure 0.1 bar. The obtained rejection efficiency fulfils the standard for treated oily wastewater discharge. It is also noteworthy that the obtained permeation flux is high for a flat sheet membrane compared to the ones reported in literature. However, based on the total fouling ratio study, the membrane with 2.0 pphr GO faced a serious fouling problem and normal rinsing it not sufficient to recover its performance. The problem could be solved with the backflushing method. These results provide a window to consider crosslinked elastomeric materials for membrane fabrication using the cost effective and environmentally friendly latex compounding method.

## Figures and Tables

**Table 1:** Formulation for membrane production.

Sample Designation	NBR	NBR-GO 0.5	NBR-GO 1.0	NBR-GO 1.5	NBR-GO 2.0
Materials	(pphr)				
NBR	100	100	100	100	100
ZnO	1.0	1.0	1.0	1.0	1.0
ZDEC	1.0	1.0	1.0	1.0	1.0
ZMBT	0.5	0.5	0.5	0.5	0.5
GO	0	0.5	1.0	1.5	2.0
KOH	1.0	1.0	1.0	1.0	1.0
Sulfur	1.0	1.0	1.0	1.0	1.0

**Table 2:** Crosslink density of the membranes.

GO Loading (pphr)	Crosslink Density (×10^−4^ mol/cm^3^)
0	9.1037 ± 0.0038
0.5	9.0525 ± 0.0018
1.0	9.2729 ± 0.0045
1.5	9.1366 ± 0.0063
2.0	9.0754 ± 0.0025

## Data Availability

Not applicable.
